# Single-catheter radiofrequency pulmonary vein isolation for atrial fibrillation: a comparative evaluation

**DOI:** 10.3389/fcvm.2026.1832133

**Published:** 2026-06-15

**Authors:** Alessio Falasca Zamponi, Fariborz Tabrizi, Anders Englund, Sergej Scheel, Jens Olson, Ivar Skoger, Christian Basile, Raffaele Scorza

**Affiliations:** 1 Capio Arytmicenter Stockholm AB, Stockholm, Sweden; 2 Department of Clinical Science and Education, Division of Cardiology, Karolinska Institutet, South Hospital, Stockholm, Sweden; 3 ANMCO Research Center, Heart Care Foundation, Florence, Italy

**Keywords:** atrial fibrillation, catheter ablation, CLOSE protocol, mapping catheter, pulmonary vein isolation

## Abstract

**Background:**

Circular mapping catheters (CMC) are used to confirm pulmonary vein isolation (PVI) in radiofrequency (RF) ablation for atrial fibrillation (AF), but may be unnecessary in CLOSE-style point-by-point workflows. We evaluated whether omitting the CMC preserves efficacy and improves efficiency in first-time PVI.

**Methods:**

We conducted a single-center, hybrid prospective–retrospective comparison of consecutive first-time PVI cases. A CMC-guided cohort (*n* = 81) underwent point-by-point RF PVI; operators were blinded to CMC electrograms during ablation and were unblinded after PVI had been verified with the ablation catheter. A subsequent CMC-free cohort (*n* = 245) used ablation-catheter–only verification. The primary endpoint was 12-month freedom from atrial arrhythmia (≥30 s) after a 90-day blanking period; Cox and negative binomial models were applied. Procedural metrics were secondary endpoints.

**Results:**

CMC-free cases had shorter procedures [105 (90, 120) vs. 120 (110, 135) min, *p* < 0.001], lower fluoroscopy [4 (3, 6) vs. 6 (5, 9) min, *p* < 0.001], and lower radiation dose [150 (96, 254) vs. 220 (167, 399) cGy·cm^2^, *p* < 0.001]. RF time and energy were reduced [2,083 (1,806, 2,493) vs. 2,343 (1,977, 2,611) s, *p* = 0.026; 71,582 (60,527, 85,399) vs. 77,335 (68,009, 87,376) J, *p* = 0.035]. Twelve-month efficacy was comparable (adjusted HR: 1.03, 95% CI: 0.58–1.84; *p* = 0.92); recurrences did not differ (adjusted IRR: 0.61, 95% CI: 0.30–1.21; *p* = 0.16). On post-ablation unblinding in the CMC-guided arm, residual PV conduction was detected in 11/321 veins (3.4%).

**Conclusion:**

In CLOSE-style first-time RF PVI, a CMC-free, single-catheter workflow was associated with improved procedural efficiency and no significant difference in observed 12-month arrhythmia outcomes. These findings are hypothesis-generating. Prospective randomized studies are needed to confirm the safety and efficacy of this approach.

## Introduction

Atrial fibrillation (AF) is the most common arrhythmia requiring intervention due to its association with significant symptoms, impaired quality of life, and increased risk of stroke (1–4). Catheter ablation is a well-established and effective therapeutic option for patients with symptomatic AF. The cornerstone of AF ablation, particularly with radiofrequency (RF) energy, is the electrical isolation of the pulmonary veins (PVs), as ectopic triggers originating within these veins play a key role in initiating and sustaining AF episodes (5).

Over time, the target of RF lesions has shifted from the PV ostia to the antral region, producing broader circumferential ablation (5). While this strategy has improved outcomes, it may increase the risk of residual gaps along lesion sets (6). To address this, operators commonly confirm pulmonary vein isolation (PVI) using a multipolar mapping catheter (MMC). Historically, the most commonly used MMC for this purpose has been a variable-loop circular mapping catheter (CMC). Positioned at the PV ostium/antrum, the CMC records PV potentials before ablation and confirms isolation by demonstrating entrance and exit block—i.e., the absence of PV electrograms and failure of pacing within the vein to capture the atrium—after wide-antral encirclement of each ipsilateral PV pair (7).

Advances in ablation technology have enhanced lesion placement accuracy, making first-pass PVI increasingly common in high-volume centers (8). As a result, the routine need for a multipolar catheter solely to confirm PVI could be reconsidered.

Eliminating the CMC from RF ablation procedures offers several potential benefits. Reducing the number of catheters in the left atrium (LA) could lower the risk of complications such as stroke and cardiac tamponade, minimize fluoroscopy time, and decrease procedural costs. A streamlined approach may reduce procedure time without compromising safety or effectiveness, making RF ablation for AF more accessible and efficient.

Prior studies have suggested that single-catheter or CMC-free RF PVI workflows are feasible and may improve procedural efficiency without an evident loss of effectiveness (9–11).

Accordingly, our study was designed to evaluate whether routine CMC use remains necessary in contemporary CLOSE-style RF PVI. We compared two consecutive single-center cohorts treated with either a CMC-guided or a CMC-free workflow. Importantly, both cohorts were treated using the same ablation catheter platform, three-dimensional mapping system, and Ablation Index (AI)–guided lesion strategy, thereby minimizing technological confounding in the comparison. In addition, the CMC cohort incorporated a single-blinded verification step, in which operators first assessed pulmonary vein isolation using the ablation catheter alone and were unblinded to the CMC only thereafter. This design allowed us not only to compare procedural efficiency and 12-month clinical outcomes between strategies, but also to provide more robust evidence regarding the incremental diagnostic contribution of routine CMC assessment after first-pass CLOSE-style isolation.

## Methods

### Study design and patient population

We conducted a single-center, hybrid prospective–retrospective comparative cohort study evaluating two consecutive calendar cohorts treated at our center. First, 81 consecutive patients were prospectively enrolled into the CMC arm between June 2020 and November 2020. In this cohort, a CMC (LASSO™, Biosense Webster) was used, but operators remained blinded to CMC signals during wide-antral encirclement and were unblinded only to confirm entrance and exit block after first-pass isolation. After completion of this prospective phase and a short transition period to implement the new workflow, we enrolled a retrospective CMC-free cohort of 245 consecutive patients treated between January 2021 and December 2021 at the same center. In this cohort, the CMC was intentionally omitted and PVI was verified with the ablation catheter alone. Allocation was non-randomized and determined by calendar time; consecutive inclusion in both periods was used to minimize selection bias. Eligibility criteria and periprocedural management were identical across cohorts: symptomatic paroxysmal or persistent AF undergoing first-time PVI; key exclusions included prior LA ablation, LA thrombus, other sustained atrial arrhythmias as a primary target of the index ablation (e.g., typical cavotricuspid isthmus–dependent right atrial flutter or left atrial flutter), and contraindications to transseptal access or anticoagulation. Informed patient consent was obtained. The study was approved by the Swedish National Ethics Committee (Dnr 2024-07893-02) and complied with the Declaration of Helsinki.

### Ablation procedure

Patients underwent ablation while maintaining uninterrupted oral anticoagulation therapy. For those with a CHA_2_DS_2_-VASc score of 1 or higher, or who presented with AF at the time of the procedure, transesophageal echocardiography (TEE) was performed to exclude the presence of intracardiac thrombus. Antiarrhythmic drugs (AAD) were discontinued at least five half-lives before the procedure, except for amiodarone, which was stopped 30 days prior. Procedures were performed at a high-volume electrophysiology center by three senior electrophysiologists; each operator had performed ≥500 AF ablations prior to study initiation, and no learning-curve effect was anticipated. All procedures were carried out under conscious sedation. Venous access was established through three right femoral venous punctures in the CMC arm and two punctures in the CMC-free arm. A decapolar catheter was advanced into the coronary sinus (CS). Transseptal puncture (TSP) was conducted using an SL1 sheath (Abbott Laboratories, Chicago, IL, USA) and a BRK1 needle (Abbott Laboratories, Chicago, IL, USA) under fluoroscopic guidance, which was standard practice at our center during the study period; contrast injection was used to confirm successful passage through the interatrial septum. Following TSP, Heparin was administered at a dose of 100 IU/kg, aiming to achieve an activated clotting time (ACT) of over 300 s. In the CMC arm, a second guidewire and SL1 sheath were advanced into the LA through the same puncture into the LA; in the CMC-free arm, only a single transseptal sheath was used. In the CMC arm, a CMC (LASSO™, Biosense Webster, Inc) was then advanced through the second SL1 sheath. The operator was blinded to CMC signals from the beginning of the procedure.

For patients presenting in AF, electrical cardioversion was performed to restore sinus rhythm prior to LA mapping. A three-dimensional navigation system (CARTO 3, Biosense Webster, Inc) was used to create a three-dimensional electroanatomical map (3D-EAM) of the LA. Respiratory artifacts were compensated byusing AccuResp Module. A deflectable sheath was used at the discretion of the operator.

The 3D-EAM was generated in sinus rhythm using a Thermocool SmartTouch SurroundFlow (STSF) catheter with an irrigated, contact-force-sensing (CF) tip (Biosense Webster, Inc).

PVI was achieved using point-by-point RF ablation to create a wide-area circumferential ablation (WACA) around each PV pair.

RF applications were delivered at a distance of at least 10 mm from the PV ostia, with power set to control temperature (maximum of 48 °C), using a maximum power output of 35 W, a targeted CF range of 3–30 g, and a saline irrigation rate of 17 mL/min. Ablations followed the CLOSE protocol, with automated lesion tagging (VisiTag™, Biosense Webster, Inc) used to mark each RF application, set to a lesion display size of 4 mm. The VisiTag™ parameters were configured as follows: minimum application time of 5 s, maximum range of 3 mm, minimum CF of 3 g, and force-over-time threshold of 30%. Intertag distance (ITD) was kept ≤6 mm. Each RF lesion was guided by an AI target of ≥400 on the posterior wall and ≥550 on the anterior wall.

After the operator assessed that the ablation lines were complete and that PVI was achieved—verified through entrance- and exit block with the ablation catheter within the PVs—the operator was then unblinded to the CMC signals. Signals from each PV were analyzed to confirm isolation. If the CMC confirmed complete isolation of all PVs, the procedure was concluded. If any residual PV signals were detected, additional ablation was performed to achieve full isolation.

In the CMC-free arm, no CMC was used. PVI was verified with the ablation catheter alone using standard criteria for entrance and exit block, including differential pacing and maneuvers to exclude far-field capture. Additional ablation was applied as needed to achieve block. Apart from the omission of the CMC and the associated blinding/unblinding step, all other procedural elements—including mapping platform, catheter technology and lesion-delivery parameters—were identical between cohorts.

### Safety outcomes

Major complications were defined as adverse events resulting in death, permanent injury, need for invasive intervention, or prolongation of hospitalization. These included cardiac tamponade requiring pericardiocentesis or surgery, stroke or transient ischemic attack, major vascular complication requiring intervention or transfusion, clinically significant air embolism, myocardial infarction, atrioesophageal fistula, or procedure-related death. Major complications were identified from procedural documentation and postprocedural medical-record review.

### Patient follow-up

Patient follow-up was conducted according to local protocols, with scheduled clinical evaluations planned at approximately 3 and 12 months after ablation, with additional assessments performed as clinically indicated. Rhythm monitoring was based on scheduled ECG evaluation at these follow-up visits, together with additional symptom-driven ECG recordings when indicated. Outcomes and rhythm documentation were collected through systematic review of the Swedish National Patient Overview (Nationell Patientöversikt, NPÖ) together with records from the study center. Recurrence of atrial arrhythmias was defined as any atrial fibrillation, atrial flutter, or atrial tachycardia episode lasting ≥30 s documented by 12-lead ECG, after a 90-day blanking period.

### Statistical analysis

All statistical analyses were carried out in R, version 4.5.0 (R Foundation for Statistical Computing, Vienna, Austria). Patient characteristics at index date were presented as frequencies (percentages) if categorical, and medians [interquartile range (IQR)] if continuous, and compared by *χ*^2^ test and Kruskal–Wallis test, respectively. The associations between treatment assignment and time-to-event outcomes were assessed by univariable and multivariable Cox proportional hazards (PH) regression models, and results were presented as hazard ratios (HR) with 95% confidence intervals (CI). The proportional hazards assumption was investigated using the scaled Schoenfeld residuals and was met. Recurrent events were modelled using a negative binomial (NB) regression including the log of time as an offset in the model and were presented as incidence rate ratios (IRR) with 95% CI. For all the evaluated outcomes a blanking period of 90 days was applied. Survival functions were visualized by the Kaplan–Meier method.

## Results

### Patient population

Patient characteristics (Table 1) show that both cohorts were predominantly male, with paroxysmal AF as the prevailing phenotype and a generally low comorbidity burden.

**Table 1:** Baseline characteristics.

Variable	CMC group (*n* = 81)	CMC-free group (*n* = 245)	*P* value
Age, years	62 [53, 67]	61.00 [55, 69]	0.60
Sex (male), *n* (%)	67 (82.7)	190 (77.6)	0.41
BMI	27 [25, 30]	27 [25, 29]	0.30
Hypertension, *n* (%)	28 (34.6)	77 (31.4)	0.70
Diabetes mellitus, *n* (%)	1 (1.2)	7 (2.9)	0.69
Stroke/TIA, *n* (%)	1 (1.2)	6 (2.4)	0.83
AF Paroxysmal/persistent, %	44/37 (54.3/45.7)	153/92 (62.4/37.6)	0.11
AF Longstanding persistent, *n* (%)	8 (9.9)	10 (4.1)	0.09
CHA2DS2-VA score	1 [0, 1]	1 [0, 2]	0.83
Cardiomyopathy, *n* (%)			
Ischaemic	3 (3.7)	15 (6.1)	0.58
Non-ischaemic	4 (4.9)	7 (2.9)	0.48
Tachycardiomyopathy	3 (3.7)	4 (1.6)	0.37
LVEF ≥50%, *n* (%)	77 (95.1)	229 (93.5)	0.80
Class I or III AAD, *n* (%)	55 (67.9)	184 (75.1)	0.18
CIED, *n* (%)	2 (2.5)	5 (2.0)	1.0

For quantitative variables, values are expressed as median [IQR]. Categorical variables are presented as counts and percentages. BMI, body mass index; TIA, transient ischaemic attack; AF, atrial fibrillation; CHA2DS2-VA, congestive heart failure, hypertension, age ≥ 75 (doubled), diabetes, stroke (doubled), vascular disease, age 65 to 74; LVEF^,^ left ventricular ejection fraction; AAD, anti-arrhythmic drugs; CIED, cardiac implantable electrical devices.

### Procedural details

Compared with the CMC-guided arm, the CMC-free cohort had shorter procedures [105 (90, 120) vs. 120 (110, 135) min, *p* < 0.001], less fluoroscopy [4 (3, 6) vs. 6 (5, 9) min, *p* < 0.001], and a lower radiation dose [150 (96, 254) vs. 220 (167, 399) cGy·cm^2^, *p* < 0.001]. Delivered RF ablation time and cumulative energy were also reduced in the CMC-free arm [2,083 (1,806, 2,493) vs. 2,343 (1,977, 2,611) s, *p* = 0.026; 71,582 (60,527, 85,399) vs. 77,335 (68,009, 87,376) J, *p* = 0.035]. In the CMC arm, upon unblinding, residual PV conduction was seen in 10 of 81 patients (12.3%), and 11 of 321 veins (3.4%) on a per-vein basis. A single periprocedural major complication (cardiac tamponade) occurred in the CMC-free cohort (1/245; 0.4%), whereas no major complications were observed in the CMC cohort (0/81). The between-group difference was not statistically significant (*p* = 1.00). Procedure-related details are summarized in Table 2.

**Table 2:** Procedural characteristics.

Variable	CMC group (*n* = 81)	CMC-free group (*n* = 245)	*P* value
Procedural time, min	120 [110, 135]	105 [90, 120]	<0.001
FT, min	6 [5, 9]	4 [3, 6]	<0.001
DAP, cGy cm^2^	220 [167, 399]	150 [96, 254]	<0.001
RF time, s	2,343 [1,977, 2,611]	2,083 [1,806, 2,493]	0.026
Energy delivered, J	77,335 [68,009, 87,376]	71,582 [60,527, 85,399]	0.035
Same day discharge, *n* (%)	11 (13.6)	12 (4.9)	0.017
PV anatomy, *n* (%)			
4 separate veins	77 (95.1)	232 (95.1)	1.00
LCO	2 (2.5)	11 (4.5)	0.53
Right intermediate	2 (2.5)	1 (0.4)	0.16

For quantitative variables, values are expressed as median [IQR]. Categorical variables are presented as counts and percentages. Statistically significant comparisons are indicated in bold. FT, fluoroscopy time; DAP, dose area product; RF, radiofrequency; PV, pulmonary vein; LCO, left common ostium.

### Clinical outcomes

**Figure 1:**
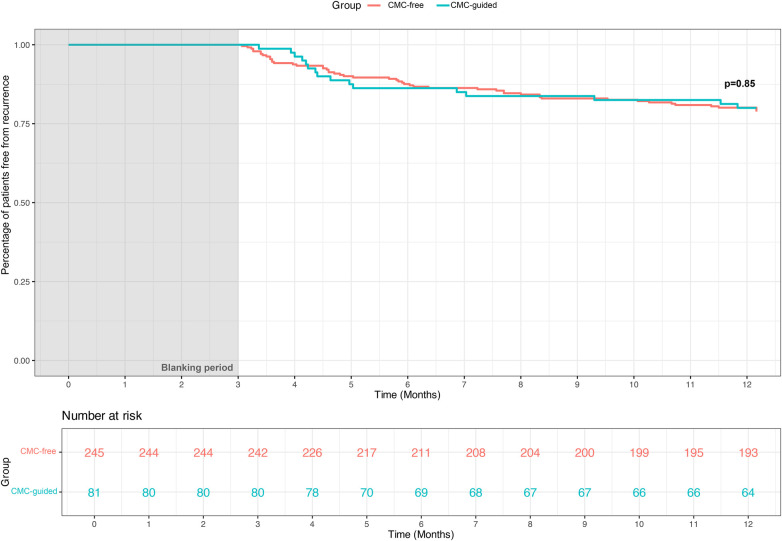
Kaplan–Meier estimates of freedom from atrial arrhythmia recurrence through 12 months after the index pulmonary vein isolation, comparing a circular-mapping-catheter (CMC)–guided strategy (*n* = 81) with a CMC-free strategy (*n* = 245).

Univariable and multivariable Cox-PH showed no significant differences in 12-month freedom from a first episode of atrial arrhythmias between strategies (Univariable HR: 0.98, 95% CI: 0.57–1.70; *p* = 0.95; Multivariable HR: 1.03, 95% CI: 0.58–1.84, *p* = 0.92). The absolute 12-month recurrence rates were 21.0% (95% CI: 11.6–29.4) in the CMC-guided group and 22.4% (95% CI: 17.0–27.5) in the CMC-free group (Figure 1). When total number of episodes of atrial arrhythmias was examined, no significant difference emerged in univariable (IRR: 0.86, 95% CI: 0.44–1.67, *p* = 0.65) or multivariable (IRR: 0.61, 95% CI: 0.30-1.21, *p* = 0.16) NB analysis.

**Figure 2:**
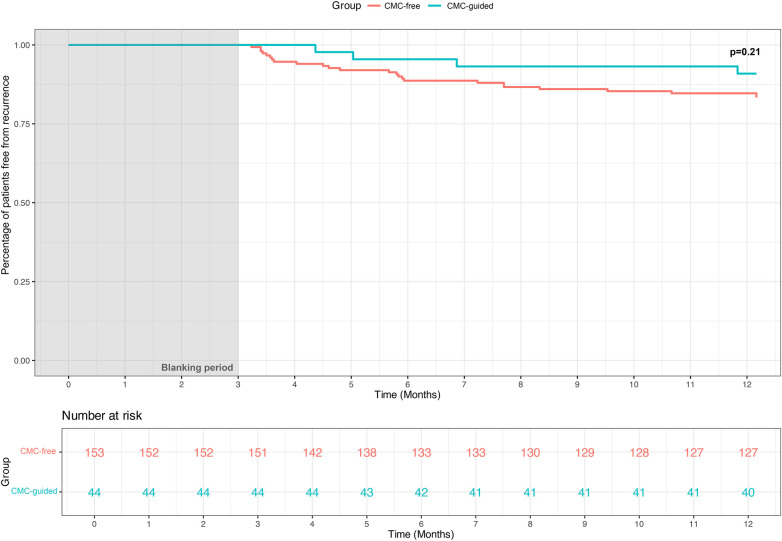
Paroxysmal AF subgroup. Kaplan–Meier curves for time to first atrial arrhythmia recurrence through 12 months after the index procedure.

**Figure 3:**
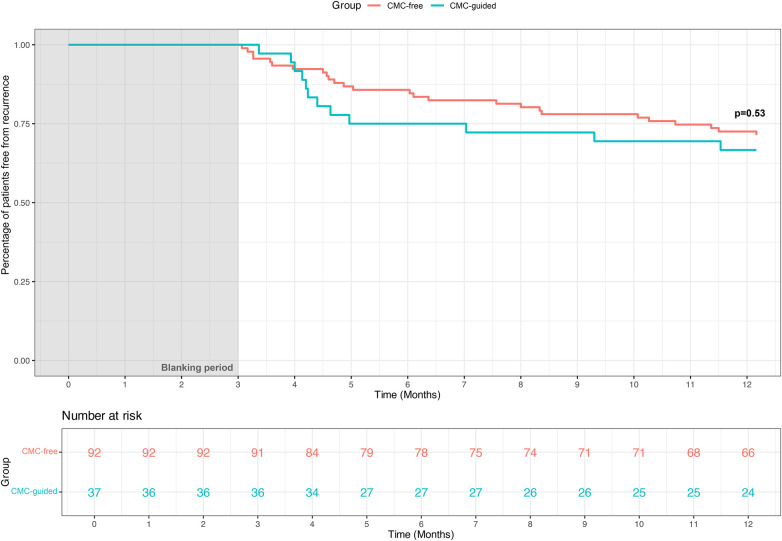
Persistent AF subgroup. Kaplan–Meier curves for time to first atrial arrhythmia recurrence through 12 months after the index procedure.

Results were similar when analyzed by AF type, showing no significant interaction neither in the Cox PH model (p-int = 0.18) or in the NB regression analysis (p-int = 0.22). Among patients with paroxysmal AF, the absolute 12-month recurrence rates were 9.1% (95% CI: 0.2–17.2) in the CMC-guided group and 18.3% (95% CI: 11.9–24.2) in the CMC-free group; among patients with persistent AF, the corresponding rates were 35.1% (95% CI: 17.8–48.8) and 29.3% (95% CI: 19.4–38.1), respectively (Figure 2, 3).

## Discussion

In this single-center, hybrid prospective–retrospective cohort, a CMC-free RF PVI workflow achieved 12-month arrhythmia outcomes comparable to a CMC-guided strategy while improving procedural efficiency. These findings are consistent with prior reports of single-catheter or CMC-free RF ablation workflows (9–11). The principal contribution of the present study lies in the factthat both cohorts were treated using the same ablation catheter platform and identical AI-guided CLOSE parameters, thereby limiting technological confounding, while the CMC arm incorporated a single-blinded verification step that provides insight into the incremental diagnostic yield of routine CMC assessment after first-pass isolation.

Prior studies have already shown that streamlined RF PVI workflows can be performed efficiently without an evident loss of efficacy. Chin et al. reported favorable acute and 12-month outcomes with a CLOSE-guided single-catheter workflow (9), whereas Badertscher et al. demonstrated procedural efficiency using an AI-guided high-power short-duration approach (10). Valeriano et al. likewise found lower procedure and fluoroscopy metrics without differences in 12-month outcomes in a comparison involving a microelectrode-enabled catheter platform (11). Compared with this earlier work, the present study reduces confounding from differences in catheter technology or lesion-delivery strategy and adds a practical evaluation of how often routine CMC use changes procedural interpretation after isolation has already been established with the ablation catheter.

### Efficacy

At 12 months, recurrence-free survival was comparable between strategies and results were consistent by substrate: paroxysmal and persistent AF. Although the study was not powered for a formal non-inferiority test, these findings argue against any clinically meaningful decrement in efficacy when omitting a CMC, provided entrance/exit block is rigorously verified with the ablation catheter within a contiguity-focused lesion set.

These observations align with contemporary single-catheter studies, including a propensity-matched comparison of microelectrode-based and CMC-guided workflows, which demonstrated similar 12-month arrhythmia-free outcomes (11). In our experience, comparable efficacy was achieved without microelectrodes, using a standard STSF ablation catheter.

### Efficiency

Through our patient series, CMC-free strategy was associated with significant gains in procedural efficiency. Compared with the CMC-guided arm, the CMC-free cohort had shorter procedures, less fluoroscopy, and lower radiation dose. Delivered RF ablation time and cumulative energy were also lower in the CMC-free arm. Importantly, our single-blinded design in the CMC arm—where operators were initially blinded to CMC electrograms and unblinded only after first-pass assessment—may itself have modestly prolonged verification steps (e.g., brief pauses for unblinding and signal review). The additional time attributable specifically to this verification protocol was not separately recorded and therefore cannot be formally quantified. This feature could bias efficiency results in favor of the CMC-free arm, and the observed differences should be interpreted in that context.

However, omitting a second LA catheter and sheath removes several workflow steps (second transseptal handling, catheter exchanges, ostial signal hunting, repeated repositioning for each PV pair). This simplification reduces catheter manipulation and fluoroscopic guidance needs, shortens LA dwell time, and limits mapping/verification cycles—all consistent with the observed reductions in procedure duration, fluoroscopy time, dose, and RF delivery.

Despite shorter procedures in the CMC-free cohort, same-day discharge was more common in the CMC-guided group. This likely reflects temporal and organizational factors rather than procedural aspects alone. In particular, the CMC-guided cohort was treated early during the COVID-19 pandemic, when pressure on inpatient capacity at our center favored same-day discharge whenever feasible. Accordingly, this difference should not be interpreted as evidence of superior procedural recovery in the CMC-guided group.

In both cohorts, the 3D-EAM of the LA was created with the ablation catheter. We intentionally performed geometry-only mapping and did not routinely acquire voltage or activation maps for substrate characterization. This approach sacrifices some electroanatomical insights but favors efficiency, and it is aligned with contemporary practice in which PVI-only remains the most widely accepted strategy for both paroxysmal and persistent AF (12).

In the evolving era of pulsed field ablation, much of the procedural advantage has shifted toward single-shot workflows for straightforward index PVI. However, point-by-point RF ablation retains important value in scenarios where flexibility is required, including patients with atypical PV anatomy, the need for individualized lesion placement, or procedures in which additional substrate modification or linear ablation may be considered. In such cases, the ability to integrate mapping and lesion delivery within a streamlined RF workflow remains attractive. A CMC-free strategy may therefore help preserve the versatility of point-by-point RF ablation while reducing catheter burden, fluoroscopy, and procedural complexity.

### Safety

The present study was not powered to detect differences in rare major complications, and event rates were very low. One cardiac tamponade occurred in the CMC-free cohort, whereas no major complications were observed in the CMC cohort; therefore, no comparative safety conclusions can be drawn from these data. Any potential safety advantages of a streamlined, single-catheter strategy should therefore be regarded as hypothesized procedural considerations rather than findings demonstrated by the present study. In principle, eliminating a second transseptal sheath and avoiding LA catheter exchanges could reduce opportunities for myocardial perforation, tamponade, thromboembolism, or air ingress during equipment passage across the sheath. Omitting a CMC also avoids device-specific hazards, including snaring or entrapment in the mitral apparatus. Similar procedural-safety arguments have already been articulated by several other authors (9–11).

### Economic aspects

Although this study was not a formal economic analysis, the pattern of results strongly suggests favorable resource use with a CMC-free strategy. By omitting both the CMC and a second transseptal sheath, the CMC-free approach yields an estimated per-case device saving of 16 020 SEK at our center (14,375 SEK for the CMC and 1,645 SEK for the transseptal sheath). Applied to the 245 patients in the CMC-free cohort, this corresponds to 3,924,900 SEK (approximately 427,000 USD) in direct device costs. In addition, the shorter procedures observed in the CMC-free arm further translate into operational savings and higher throughput, supporting a more efficient lab utilization—an issue that is central to electrophysiology services facing sustained demand, staffing constraints, and post-pandemic backlogs. Importantly, while avoiding CMC costs, we achieved comparable 12-month clinical efficacy with a less expensive catheter platform, supporting a favorable budget impact for a streamlined, CMC-free, STSF-based workflow when micro-electrode catheters are not clinically required.

### Insights from the single-blinded verification step

A distinctive feature of the present study is the single-blinded CMC verification step, which allowed us to examine the incremental contribution of routine CMC use after first-pass CLOSE-style PVI had already been assessed with the ablation catheter. This is clinically relevant because, in contemporary point-by-point RF ablation, the practical value of a multipolar catheter depends not simply on whether it can record PV signals, but on whether it meaningfully changes procedural interpretation or management once a contiguous AI-guided lesion set has already been delivered. In the CMC arm, operators completed wide-antral encirclement and verified entrance and exit block with the ablation catheter while remaining blinded to CMC electrograms; the CMC was unblinded only thereafter to confirm isolation.

Although the CMC has long been regarded as a reference tool for confirming PV conduction, several anatomic and electrophysiologic features may limit its performance in this setting. WACA improves outcomes but shifts relevant breakthrough sites toward the carina and antral segments, where epicardial and interlacing myocardial sleeves can create complex, anisotropic activation and small residual gaps (13, 14). A circular catheter positioned at the ostium may therefore fail to sample the earliest activation or accurately localize conduction gaps, potentially prompting additional ostial lesions despite adequately delivered antral lesion sets. By contrast, alternative single-catheter verification strategies—such as loss of pace capture along the ablation line with adequate contact force, focused sampling within the WACA, and maneuvers to confirm entrance and exit block—have been shown to feasibly adjudicate PVI without routine multipolar catheters (15–19).

**Figure 4:**
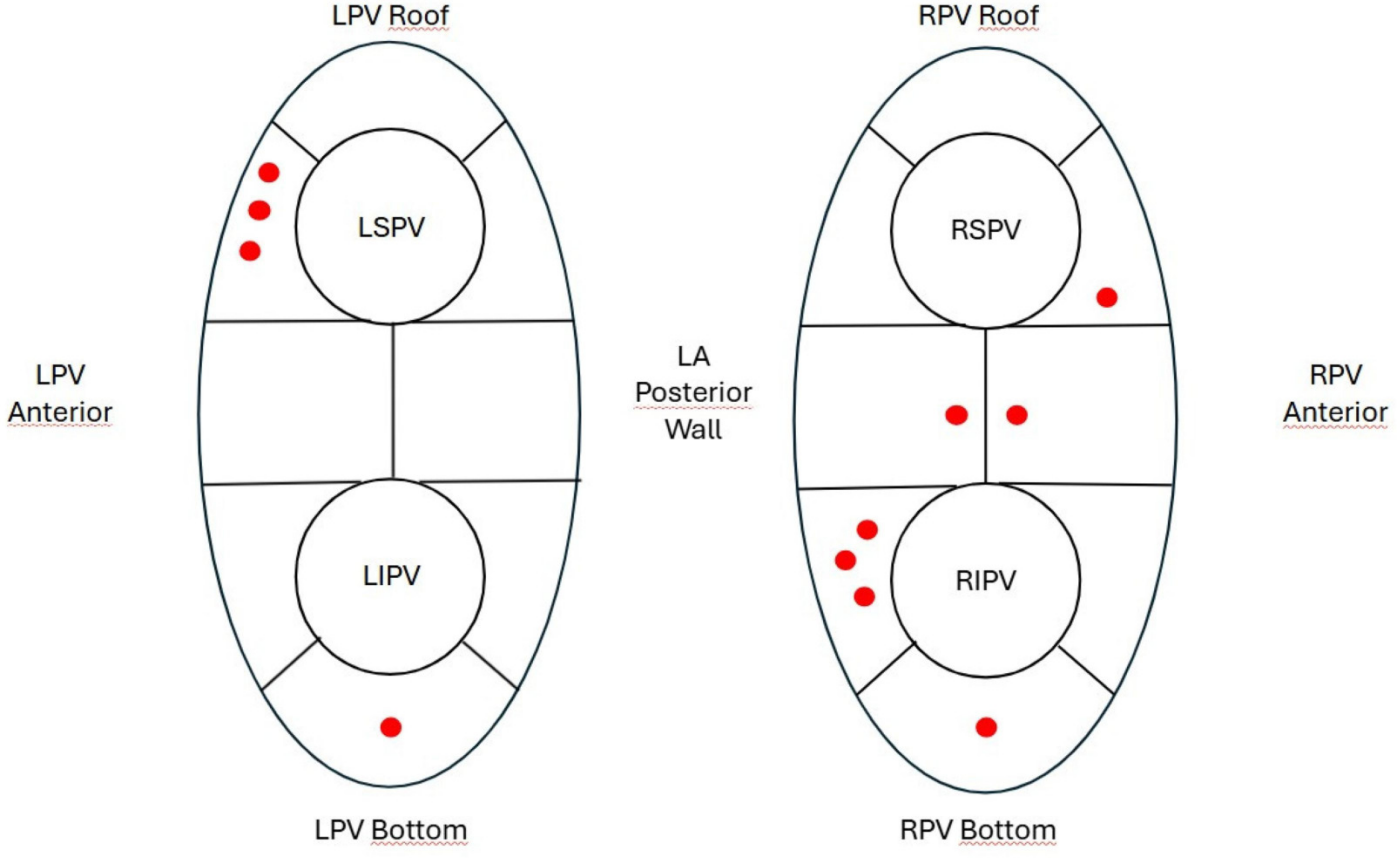
Localization of residual pulmonary vein conduction at CMC unblinding in the CMC-guided arm.

In our cohort, unblinding revealed residual PV conduction in only 10 of 81 patients (12.3%), corresponding to 11 of 321 veins (3.4%) on a per-vein basis. When present, residual signals were most often localized to the LSPV ridge and the posteroinferior RIPV (Figure 4). In these cases, additional ablation lesions were delivered to achieve complete acute isolation according to the study protocol. In a subset of cases, putative CMC signals persisted despite targeted ablation, suggesting suboptimal catheter position or orientation, or far-field recording. Conversely, residual conduction along the carina or antral line was at times identified with the ablation catheter using systematic entrance- and exit-block testing and high-output pacing while the CMC remained apparently negative. Although assisting personnel could see the CMC signals during first-pass verification, they did not communicate discrepancies to the operator until the prespecified unblinding step. Taken together, these observations suggest that after CLOSE-style first-pass isolation, the incremental diagnostic yield of routine CMC use is modest in most cases and may be constrained by incomplete coverage of antral breakthrough sites, variable tissue contact, and far-field contamination. Given the small number of cases with residual conduction detected at unblinding, the present study was not designed or powered to determine whether the additional lesions influenced longer-term follow-up outcomes.

### Limitations

Our study has several limitations. First, this was a single-center observational study in which patient allocation was non-randomized and determined by calendar time, introducing the possibility of temporal and workflow-related confounding. Although both cohorts were treated using the same ablation platform, lesion-delivery strategy, and CLOSE-style AI-guided workflow, changes over time in operator experience, procedural routines, laboratory organization, discharge practices, and institutional workflow may have influenced procedural efficiency outcomes. This is particularly relevant because the two cohorts were treated during different phases of the COVID-19 period, which may have affected organizational routines, inpatient bed pressure, and same-day discharge practices.

Second, the single-blinded verification step in the CMC-guided arm may itself have modestly prolonged procedural duration. Brief pauses for unblinding, CMC signal review, and reassessment after first-pass evaluation may therefore have biased efficiency comparisons in favor of the CMC-free workflow. The additional time attributable specifically to this verification protocol was not separately recorded and therefore cannot be formally quantified.

Third, rhythm follow-up was based primarily on scheduled ECGs and symptom-driven rhythm documentation, without systematic prolonged Holter monitoring or implantable rhythm surveillance. As a result, asymptomatic AF or atrial tachyarrhythmia recurrences may have been underestimated in both groups. This limitation may affect the absolute recurrence rates and should be considered when interpreting the comparison of 12-month rhythm outcomes.

Fourth, the study was not designed or powered as a formal non-inferiority trial. Therefore, the absence of a statistically significant difference in 12-month recurrence should not be interpreted as definitive evidence of non-inferiority of the CMC-free strategy. Rather, the efficacy findings should be interpreted as hypothesis-generating and supportive of comparable outcomes within the limitations of the observational design, sample size, and rhythm-monitoring strategy.

Finally, this study specifically evaluated the role of a traditional CMC within a CLOSE-style, AI-guided point-by-point RF workflow. Since the study period, newer multipolar mapping catheters, including higher-density grid and multispline designs with orthogonal or directional signal acquisition, have become more widely available. These platforms provide greater spatial sampling, faster activation mapping, and potentially better gap detection than traditional circular catheters (20, 21). As such, our findings should not be over-extrapolated to contemporary high-density multipolar mapping catheters. It is possible that modern mapping catheters could shift the efficiency/efficacy balance, for example by accelerating verification or by adding value in complex anatomies, redo procedures, or non-PV substrate mapping. However, our study did not test these devices, and we cannot infer their impact on outcomes, safety, or cost. Prospective, ideally randomized evaluations comparing ablation-catheter–only verification with selective or routine use of contemporary high-density mapping catheters are needed to define where advanced multipolar mapping adds net clinical benefit.

## Conclusion

In this single-center comparison of first-time CLOSE-style RF PVI, a CMC-free workflow appears feasible and with improved procedural efficiency and a low incremental diagnostic yield of routine CMC verification after first pass isolation. No significant differences in observed 12-month arrhythmia outcomes were detected; however, given the observational design and study limitations, these findings should be considered hypothesis-generating rather than a proof of clinical equivalence. Prospective, and ideally randomized, studies are warranted to determine the impact of a CMC-free strategy on long-term efficacy, safety, and resource utilization.

## Data Availability

The raw data supporting the conclusions of this article will be made available by the authors, without undue reservation.
